# Supplementary material for: Toxicity of Beauty Salon Effluents Contaminated with Hair Dye on Aquatic Organisms
Source: Toxics. 2023 Nov 7;11(11):911. doi: 10.3390/toxics11110911 (PMC10674561; doi:10.3390/toxics11110911)
Supplement: Supplementary file 1 [file toxics-11-00911-s001.zip › toxics-2571339-supplementary.pdf]

Table S1: Components of brown hair dye, shampoo and hair conditioner.

PRODUCTS		
**DYE**	**HYDROGEN PEROXIDE SOLUTION**	**SHAMPOO AND CONDITIONER**
Water	Water	Water
Cetostearyl alcohol	Hydrogen peroxide	Sodium Lauryl Ether Sulphate
Ethanolamine	Cetostearyl alcohol	Vegetable ingredient derived from coconut oil
Laurylether	Ceteareth-25	Cocamidopropyl betaine
Sodium Lauryl Ether Sulphate	Salicylic acid	Disterated cocoa
Glycerol Monostearate	Phosphoric acid	Fragrance
2,5-Diaminotoluene	Disodium phosphate	Dmdm Hydantoin
Sodium sulfate	Etidronic acid	Polyquaternium - 7
Myristyl alcohol		Sodium chloride
Sodium lauryl sulfate		Citric acid
Beeswax		Disodium EDTA
Sodium Cocoyl Isothionate		Sodium citrate
Perfume		Thickener
Shimmering / pearlescent pigment		Benzoic acid
Hydroxyethyl starch - 3,4 - methylmedioxyanililine HCl		Hydrolyzed silk
Resorcinol		Phenoxyethanol
Sodium sulfite		Cotton
M- Aminophenol		Shea Butter
Ascorbic acid		Black tea
EDTA- Disodium Phosphate		Green tea extract
hydrolyzed keratin		Chamomile
2- methylresorcinol		Shea
Titanium dioxide (CI 77891)		Cinnamon
Hexyl cinnamaldehyde		Coconut extract
Limonene		*Commiphora myrrha* (African Myrrh) resin extract
Benzyl Benzoate		Macadamia oil
Phenylenediamines (Diaminotoluenes)		Olive oil/olive oil
